# Supplementary material for: Characterization of antimalarial activity of artemisinin-based hybrid drugs
Source: Antimicrob Agents Chemother. 2024 Jun 20;68(7):e00143-24. doi: 10.1128/aac.00143-24 (PMC11232401; doi:10.1128/aac.00143-24)
Supplement: Supplemental material — Figures S1 to S7; Tables S1 to S3. [file aac.00143-24-s0001.docx]

**supplemental material**

**Characterization of antimalarial activity of artemisinin-based hybrid drugs**

Helenita Costa Quadros,^[a]^ Lars Herrmann,^[b]^ Jeanne Manaranche,^[c,d,e]^ Lucie Paloque,^[c,d,e]^ Mariana C. Borges-Silva,^[a]^ Godwin Akpeko Dziwornu,^[f]^ Sarah D’Alessandro,^[g]^ Kelly Chibale,^[f,h]^ Nicoletta Basilico,^[i]^ Françoise Benoit-Vical,^[c,d,e]*^ Svetlana B. Tsogoeva,^[b]*^ and Diogo Rodrigo M. Moreira^[a]*^

^a^ Instituto Gonçalo Moniz, Fundação Oswaldo Cruz (FIOCRUZ), Salvador 40296-710, Brazil.

^b^ Organic Chemistry Chair I and Interdisciplinary Center for Molecular Materials (ICMM), Friedrich- Alexander-Universität of Erlangen-Nürnberg, Nikolaus Fiebiger-Straße 10, 91058 Erlangen, Germany.

^c^ LCC-CNRS, Laboratoire de Chimie de Coordination, Université de Toulouse, CNRS, Toulouse, France.

^d^ MAAP, New Antimalarial Molecules and Pharmacological Approaches, Inserm ERL 1289, Toulouse, France.

^e^ Institut de Pharmacologie et de Biologie Structurale (IPBS), Université de Toulouse, CNRS, Université Toulouse III - Paul Sabatier (UPS), Toulouse, France.

^f^ Drug Discovery and Development Centre (H3D), Department of Chemistry, University of Cape Town, Rondebosch 7701, South Africa.

^g^ Dipartimento di Scienze Farmacologiche e Biomolecolari, Università degli Studi di Milano, 20133 Milan, Italy.

^h^ South African Medical Research Council Drug Discovery and Development Research Unit, Department of Chemistry and Institute of Infectious Diseases and Molecular Medicine, University of Cape Town, Rondebosch 7701, South Africa.

^i^ Dipartimento di Scienze Biomediche, Chirurgiche e Odontoiatriche, Universitá degli Studi di Milano, 20133 Milan, Italy.

*Address correspondence to Françoise Benoit-Vical, francoise.vical@inserm.fr; Svetlana Tsogoeva, svetlana.tsogoeva@fau.de; Diogo Moreira, diogo.magalhaes@fiocruz.br.

**Table of content**

| **Entry** | **Description** | **Page** |
| --- | --- | --- |
| **Figure S1** | Quantification of fluorescence of hybrid (**1**) in the supernatant and cell pellet. | 39 |
| **Table S1** | Stage-specificity of antiparasitic activity in the asexual blood stages assayed against CQ-sensitive strain NF54 of *P. falciparum*. Data associated to Figure 2A in the main text of the manuscript. | 40 |
| **Figure S2** | Exposure time dependence of antiparasitic activity against ring stages of CQ-sensitive strain NF54 of *P. falciparum*. Data associated to Figure 2B in the main text of the manuscript. | 41 |
| **Figure S3** | Speed of antiparasitic activity of hybrids (**1**) and (**2**) against CQ-sensitive NF54 strain of *P. falciparum*. | 42 |
| **Figure S4** | *In vitro* interactions of CQ and MFQ plus hybrids (**1**) and (**2**) against the NF54 strain of *P. falciparum*. | 43 |
| **Table S2** | IC_50_ of drugs alone or in combination with CQ or MFQ. | 44 |
| **Figure S5** | Data associated to Figure 3 in the main text of the manuscript. | 45 |
| **Table S3** | Summary of IC_50_ values against F32-ART strain (ART-resistant) and its isogenic laboratory control F32-TEM strain (ART-sensitive) of *P. falciparum.* Data associated to Figure 4 in the main text of the manuscript. | 46 |
| **Figure S6** | Summary of IC_50_ values derived from beta-hematin inhibitory activity (BHIA). Data associated to Figure 5 in the main text of the manuscript. | 47 |
| **Figure S7** | Quantification of heme species involved in the heme detoxification of *P. falciparum* by treatment with DHA, CQ, and hybrids. Data associated to Figure 5 in the main text of the manuscript. | 48 |

**Figure S1:** Quantification of hybrid LH70 (**1**) by fluorescence and HPLC in the uRBC-derived supernatants. Panel (A) shows the quantification of hybrid LH70 (**1**) by HPLC (Shimadzu, C18 column and UV-Vis detector set at 215 nm) in the uRBC-derived supernatants harvested at indicated incubation times. Standard represents a sample of compound freshly dissolved in DMSO and then diluted in RPMI. Panel (B) shows the quantification of hybrid LH70 (**1**) by fluorescent signal measured in a plate reader (Fluoroskan, Thermo) in the uRBC-derived supernatants harvested at indicated incubation times. Panel (C) shows the quantification of hybrid LH70 (**1**) by fluorescence of the compound freshly dissolved in pure DMSO or dissolved in DMSO and then diluted in RPMI (final concentration of DMSO 0.5% v/v). Panel (D) shows the quantification of hybrid LH70 (**1**) using a fluorescence plate reader. The drug was quantified in the supernatant of the cell culture after the second and third steps of medium replacement and washing out. In all panels, values are mean and error bars are standard deviation of one experiment performed in two replicates; black dotted line means the limit of detection. RFU = relative fluorescence units.

**Table S1**: Stage-specificity of antiparasitic activity in the asexual blood stages assayed against the CQ-sensitive strain NF54 of *P. falciparum*. Data associated to Figure 2A in the main text of the manuscript.

| **Compounds** | **NF54 strain of *P. falciparum* (IC_50,_ nM, median ± SEM)** | | | |
| --- | --- | --- | --- | --- |
|  | **Ring stages ^[a]^** | **Asynchronous ^[b]^** | **72 h^[c]^** | ***p* values^[d]^** |
| LH70 (**1**) | 1.5 ± 0.3 | 3.5 ± 0.3 | 3.0 ± 0.3 | - |
| 163A (**2**) | 9.0 ± 1.5 | 19.1 ± 1.8 | 8.1 ± 1.0 | <0.05 |
| DHA | 5.0 ± 0.5 | 4.6 ± 0.9 | 2.1 ± 0.6 | - |
| MFQ | 37.2 ± 5.2 | 61.6 ± 11.9 | 20.2 ± 1.3 | <0.05 |

^[a]^ Synchronized ring-stage parasites were incubated in the presence of compounds for 6 h, drugs were removed by washing, and plates were incubated for an additional 66 h. ^[b]^ Asynchronous parasites were incubated in the presence of compounds for 6 h, drugs were removed by washing, and plates were incubated for an additional 66 h. ^[c]^ Asynchronous parasites were incubated in the presence of compounds for 72 h. ^[a,b]^ Experiments were performed in parallel. ^[d]^ Values (rings *versus* asynchronous) were significantly different as determined by Student’s *t* test. Parasite viability was determined by parasite lactate dehydrogenase. Data are from three experiments performed in duplicate. MFQ = mefloquine; DHA = dihydroartemisinin; SEM = standard error of the mean.

| **Compounds** | **NF54 strain of *P. falciparum* (IC_50,_ nM, median ± SEM)** | | | |
| --- | --- | --- | --- | --- |
|  | **3 h + 69 h ^[a]^** | **6 h + 66 h ^[a]^** | **72 h^[b]^** | ***p* values^[c]^** |
| LH70 (**1**) | 220 ± 80 | 1.5 ± 0.3 | 1.5 ± 0.1 | N.S. |
| 163A (**2**) | 75 ± 18 | 9.0 ± 1.5 | 3.8 ± 0.4 | <0.05 |
| DHA | 100 ± 25 | 5.0 ± 0.5 | 1.9 ± 0.2 | <0.05 |
| MFQ | >600 | 37.2 ± 5.2 | 10.3 ± 2.6 | <0.01 |

**Figure S2**: Exposure time dependence of antiparasitic activity against ring stages of CQ-sensitive strain NF54 of *P. falciparum*. Data associated to Figure 2B in the main text of the manuscript. Footnotes for table: ^[a]^ Synchronized ring-stage parasites were incubated in the presence of compounds at indicated times, drugs were removed by washing, and plates were incubated until 72 h.^[b]^ Parasites were incubated in the presence of compounds for 72 h. ^[a,b]^ Experiments were performed in parallel (paired). Parasite viability was determined by parasite lactate dehydrogenase (pLDH) assay. Data are from three experiments performed in duplicate. ^[c]^ Values (6 h versus 72 h) were significantly different as determined by Student’s *t* test. MFQ = mefloquine. DHA = dihydroartemisinin; SEM = standard error of the mean; N.S. = not statistically significant.

**(A)**

**(B)**

| **Compounds** | **Asynchronous NF54 strain of *P. falciparum* (IC_50_, nM)^[a]^** | | | **Conclusion** |
| --- | --- | --- | --- | --- |
|  | **24 h** | **48 h** | **72 h** |  |
| LH70 (**1**) | 2.4 (2.1–2.7) | 3.0 (2.6–3.5) | 2.0 (1.8–2.4) | Fast-acting |
| 163A (**2**) | 9.4 (8.0–11.2) | 4.3 (3.8–5.0) | 5.6 (5.0–6.4) | Fast-acting |
| DHA | 8.3 (3.9–10) | 2.9 (2.0–4.1) | 2.7 (2.5–2.9) | Fast-acting |
| ATO | >600 | 5.3 (4.1–6.0) | 0.78 (0.39–0.91) | Slow-acting |

**Figure S3**: Speed of antiparasitic activity of hybrids (**1**) and (**2**) against CQ-sensitive NF54 strain of *P. falciparum*. Panel A: Representative response-concentration curves. Panel B: A table summarizing the results. Footnotes for table: ^[a]^ Parasite viability was accessed at each indicated time (24, 48, or 72 h) after addition of drugs. Values are the mean and 95% confidence interval of one experiment using each concentration of compounds in duplicate. Parasite viability was determined by parasite lactate dehydrogenase (pLDH) assay. DHA = dihydroartemisinin; ATO = atovaquone.

(A)

(B)

| **Combination** | **Mean ΣFIC_50_ after 72 h at a drug ratio of 1:1** | **Interaction** | **Interpretation** |
| --- | --- | --- | --- |
| (**1**) + CQ | 1.0 | Nondetrimental | Additivity |
| (**1**) + MFQ | 0.6 | Nondetrimental | Additivity |
| (**2**) + CQ | 1.5 | Detrimental | Antagonistic |
| (**2**) + MFQ | 0.9 | Nondetrimental | Additivity |

**Figure S4**. ***In vitro* interactions of CQ and MFQ plus hybrids (1) and (2) against the NF54 strain of *P. falciparum*.** Panel A shows representative isobologram plots. Values represent one experiment; each concentration of drug combination was tested in technical duplicate. Black dashed line indicates **Σ**FIC = 1 (absolute additivity). Panel B shows a table summarizing the **Σ**FIC indices derived from panel A. Values are the means of two independent experiments. **Σ**FIC = fractional inhibitory concentration. FIC index <1 is synergistic, ~1 is additive, and >1 is antagonistic. Nondetrimental interactions are 0.25 <**Σ**FIC <1.25. CQ = chloroquine; MFQ = mefloquine.

**Table S2**. IC_50_ of drugs alone or in combination with CQ or MFQ. Data associated to Figure S4.

| **Ring stages of NF54 strain *P. falciparum* (IC_50_, nM)^[a]^** | | | | |
| --- | --- | --- | --- | --- |
| **Drugs alone** | | **Drugs in combination at different ratios** | | |
|  |  | **3:1** | **1:1** | **1:3** |
| Hybrid (**1**) | CQ | (**1**):CQ | (**1**):CQ | (**1**):CQ |
| 2.2 (2.0–2.4) | 10.5 (7.5–14.8) | 1.9 (1.6–2.1) | 1.8 (1.5–2.2) | 2.0 (1.7–2.3) |
| Hydrid (**1**) | MFQ | (**1**):MFQ | (**1**):MFQ | (**1**):MFQ |
| 2.0 (1.9–2.2) | 10.3 (8.6–12.3) | 1.9 (1.7–2.0) | 1.5 (1.3–1.7) | 2.5 (2.0–3.1) |
| Hybrid (**2**) | CQ | (**2**):CQ | (**2**):CQ | (**2**):CQ |
| 4.7 (4.6–4.8) | 10.4 (9.2–11.7) | 4.9 (4.5–5.2) | 4.8 (4.4–5.1) | 4.4 (3.7–5.1) |
| Hybrid (**2**) | MFQ | (**2**):MFQ | (**2**):MFQ | (**2**):MFQ |
| 4.8 (4.5–5.1) | 17.0 (14.0–20.6) | 4.0 (3.8–4.3) | 3.4 (2.8–4.1) | 2.5 (2.0–2.9) |

**^[a]^** Values are the mean and 95% confidence interval of one experiment for each concentration of compounds in duplicate. CQ = chloroquine; MFQ = mefloquine.

(A)

(B)

| **Compounds** | **IC_50_ (nM) of supernatants harvested at indicated time from uRBC (1.0% hematocrit) against 3D7 strain of *P. falciparum* ^[a]^** | | | **Fold changes**  **(24 h/0.16 h) ^[c]^** |
| --- | --- | --- | --- | --- |
|  | **0.16 h** | **6 h** | **24 h** |  |
| LH70 (**1**) | 1.8 ± 0.33 | 1.8 ± 0.44 | 1.9 ± 0.53 | 1.0 ± 0.23 |
| 163A (**2**) | 3.4 ± 0.33 | 3.9 ± 0.40 | 4.1 ± 1.0 | 1.1 ± 0.31 |
| DHA | 4.5 ± 1.1 | 5.7 ± 0.8 | 80.4 ± 10.0** | 17.0 ± 3.1 |
| AQ ^[d]^ | 3.9 (3.6–4.1) | 3.4 (3.1–3.7) | 3.0 (2.90–3.3) | 0.76 |
| **Compounds** | **IC_50_ (nM) of supernatants harvested at indicated time from uRBC (2.5% hematocrit) against 3D7 strain of *P. falciparum* ^[a]^** | | | **Fold changes**  **(24 h/0.16 h) ^[c]^** |
| LH70 (**1**) | 1.3 ± 0.44 | 1.5 ± 0.63 | 4.8 ± 1.1 | 3.6 ± 0.8 |
| 163A (**2**) | 2.8 ± 0.51 | 4.3 ± 0.74 | 29.0 ± 6.9** | 10.7 ± 4.1 |
| DHA | 6.7 ± 2.5 | 22.7 ± 3.1* | 147.3 ± 10.3** | 25.9 ± 4.7 |
| AQ ^[d]^ | 3.6 (3.3–4.3) | 3.8 (3.5–4.0) | 3.3 (2.2–3.4) | 0.91 |
| **Compounds** | **IC_50_ (nM) of supernatants harvested at indicated time from uRBC (1.0% hematocrit) against D10 strain of *P. falciparum* ^[b]^** | | | **Fold changes**  **(24 h/0.16 h) ^[c]^** |
| LH70 (**1**) | 1.8 ± 0.08 | 1.4 ± 0.19 | 1.6 ± 0.22 | 0.90 ± 0.08 |
| 163A (**2**) | 3.7 ± 1.32 | 3.9 ± 0.31 | 4.3 ± 0.06 | 1.25 ± 0.46 |
| DHA | 3.5 ± 0.43 | 6.6 ± 0.30 | 70.0 ± 8.92** | 19.9 ± 0.09 |
| **Compounds** | **IC_50_ (nM) of supernatants harvested from uRBC (1.0% hematocrit) against W2 strain (CQ-resistant) of *P. falciparum* ^[b]^** | | | **Fold changes**  **(24 h/0.16 h) ^[c]^** |
| LH70 (**1**) | 1.9 ± 0.13 | 1.5 ± 0.39 | 1.9 ± 0.53 | 0.98 ± 0.22 |
| 163A (**2**) | 4.3 ± 1.74 | 4.1 ± 1.38 | 5.8 ± 2.27 | 1.38 ± 0.15 |
| DHA | 1.9 ± 0.25 | 3.73 ± 1.20 | 31.4 ± 8.02** | 15.82 ± 2.21 |

**Figure S5**: Data associated to Figure 3 in the main text of the manuscript. Panel A: Representative response-concentration curves of the antiplasmodial activity of supernatant-derived uRBC (2.5% hematocrit) harvested at indicated times (0.16, 6, and 24 h). Drug activity on parasite viability was determined 72 h after parasite incubation (3D7 strain, asynchronous). Values were subtracted from control (without treatment), transformed, and normalized to calculate nonlinear fit. Parasite viability was determined by SYBR Green I. Values are mean and error bars are the standard deviation of a single experiment, each concentration in duplicate. Panel B: A table summarizing the IC_50_ values. Footnotes: ^[a]^ Parasite viability was determined by SYBR Green I. ^[b]^ Parasite viability was determined by pLDH readout. Values are median ± SEM of three independent experiments, each concentration in duplicate. ^[c]^ Calculated as the ratio of IC_50_ at 24 h to IC_50_ at 0.16 h.^[d]^ These values are expressed as mean and 95% confidence interval (CI) of one experiment, each concentration in duplicate. AQ = amodiaquine; DHA = dihydroartemisinin; uRBC = uninfected red blood cells. Unpaired two-tailed *t*-test at 95% CI: **p* <0.05; ***p* <0.01.

**Table S3**: Summary of IC_50_ values against F32-ART strain (ART-resistant) and its isogenic laboratory control F32-TEM strain (ART-sensitive) of *P. falciparum.*

| **Compounds** | **IC_50_ (nM, mean ± SD) for *P. falciparum* (48 h)** | |
| --- | --- | --- |
|  | F32-TEM (ART-sensitive) | F32-ART (ART-resistant) |
| DHA | 2.0 ± 0.2 | 0.8 ± 0.8 |
| LH70 (**1**) | 2.6 ± 0.6 | 4.2 ± 1.3 |
| 163A (**2**) | 4.8 ± 1.2 | 4.5 ± 2.5 |

^[a]^ Parasites were exposed for 48 h of incubation in the presence of drugs, then parasite viability was determined using SYBR Green I. Mean of two independent experiments. DHA = dihydroartemisinin; SD = standard deviation.

| **Drugs** | **IC_50_ (μM) values of R-BHIA using heme (iron II) ^[a]^** | | | **O-BHIA hematin**  **(iron III)^[b]^** |
| --- | --- | --- | --- | --- |
|  | **10 minutes** | **120 minutes** | **Fold changes ^[c]^** |  |
| LH70 (**1**) | 609 (473.8–782) | 284 (258–314) | 2.1 | >1000 |
| DHA | 262 (221–304) | 161 (133–197) | 1.6 | >1000 |
| CQ | 189 (167.3–216) | 222 (187–261) | 0.85 | 228 (208.5–247) |

**Figure S6:** A summary of IC_50_ values derived from β-hematin inhibitory activity (BHIA). Footnotes for table: ^[a,b]^ Values are mean and 95% confidence interval of one experiment, each concentration in triplicate. ^[a]^ Using heme (Fe[II]PPIX[Cl]) as a starting reagent. ^[b]^ Using hematin (Fe[III]PPIX[OH]) as a starting reagent. ^[c]^ Ratio of IC_50_ (10 minutes) to IC_50_ (120 minutes). CQ = chloroquine. R-BHIA = reducing β-hematin inhibitory activity. O-BHIA = oxidizing β-hematin inhibitory activity.

**Figure S7**: Quantification of heme species involved in the heme detoxification of *P. falciparum* by treatment with DHA, CQ, and hybrids. Values are the mean and error bars are the standard deviation from two independent experiments, each one using two biological replicates. CQ = chloroquine. DHA = dihydroartemisinin. Data associated to Figure 5 in the main text of the manuscript.
